# Desmoid Fibromatosis of the Gastric Wall Mimicking Needle Tract Seeding after Endoscopic Ultrasound‐Guided Fine‐Needle Biopsy for Pancreatic Cancer: A Case Report

**DOI:** 10.1002/deo2.70336

**Published:** 2026-04-28

**Authors:** Kazuya Koyama, Kenji Takahashi, Hidemasa Kawabata, Yusuke Ono, Nobue Tamamura, Tetsuhiro Okada, Hidetaka Iwamoto, Mishie Tanino, Mikihiro Fujiya, Yusuke Mizukami

**Affiliations:** ^1^ Department of Internal Medicine Division of Gastroenterology Asahikawa Medical University Hokkaido Japan; ^2^ Center For Intractable Diseases and ImmunoGenomics National Institutes of Biomedical Innovation Health and Nutrition Osaka Japan; ^3^ Institute of Biomedical Research Sapporo Higashi Tokushukai Hospital Hokkaido Japan; ^4^ Department of Diagnostic Pathology Asahikawa Medical University Hospital Hokkaido Japan

**Keywords:** CTNNB1, desmoid fibromatosis, EUS‐FNB, needle tract seeding, pancreatic cancer

## Abstract

Endoscopic ultrasound‐guided fine‐needle biopsy (EUS‐FNB) is widely used for the pathological diagnosis of pancreatic diseases. However, needle tract seeding (NTS) remains a clinical concern, particularly when the puncture route is not included in the surgical resection field. We report a case in which distinguishing NTS from other pathologies was challenging. A woman in her 70s underwent EUS‐FNB via the gastric antrum for a 10‐mm pancreatic head lesion, which was diagnosed as adenocarcinoma (cT1bN0M0, Stage IA). She subsequently underwent pancreaticoduodenectomy (pT3N0M0, Stage IIA) without adjuvant chemotherapy. Two years postoperatively, a 30‐mm submucosal tumor was detected in the stomach. Although positron emission tomography‐computed tomography demonstrated fluorodeoxyglucose uptake, three separate sessions of EUS‐FNB failed to yield a definitive diagnosis, with NTS being strongly suspected. Partial gastrectomy was performed. Histopathology revealed a fascicular proliferation of spindle‐shaped cells with nuclear β‐catenin positivity, leading to the diagnosis of a desmoid fibromatosis (DF). This case underscores that although NTS should be considered when a lesion arises along the EUS‐FNB puncture tract, alternative diagnoses, including DF, must also be carefully evaluated.

## 1 Introduction

In recent years, the use of neoadjuvant chemotherapy and comprehensive genomic profiling has increased, which has increased the importance of obtaining tissue samples before initiating treatment for pancreatic cancer. EUS‐FNB has consequently become a standard modality [1]. However, needle tract seeding (NTS) remains a clinically significant concern, particularly when the puncture tract is not included in the surgical resection field, with its frequency in pancreatic body/tail cancer reported to be about 3.4% [2]. Here, we report a case of desmoid fibromatosis (DF) arising along the endoscopic ultrasound‐guided fine‐needle biopsy (EUS‐FNB) puncture tract in the gastric wall after surgical resection of a pancreatic head cancer, which posed a diagnostic challenge in distinguishing from NTS.

## 2 Case Report

**FIGURE 1:**
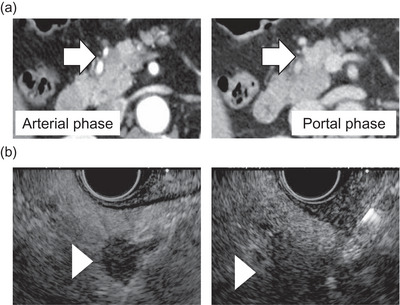
(a) Arrows (⇒) indicate primary pancreatic cancer in a CT scan. (b) Preoperative EUS‐FNB for pancreatic tumor. Arrowheads (▷) indicate primary pancreatic cancer. CT, computed tomography; EUS‐FNB, Endoscopic ultrasound‐guided fine‐needle biopsy.

A 70‐year‐old Japanese woman with no specific symptoms presented to the neurology department for follow‐up after a stroke. Her medical history included fatty liver, dyslipidemia, and hypertension; notably, she had no family history of familial adenomatous polyposis (FAP) and no history of anti‐estrogen therapy. She was referred to the gastroenterology department for evaluation of liver enzyme levels. Computed tomography (CT) revealed a 10‐mm hypovascular mass in the pancreatic head without lymph node involvement or distant metastasis (Figure 1a).

EUS (GF‐UCT260; Olympus Medical Systems, Tokyo, Japan) showed a well‐defined, hypoechoic 9.7‐mm lesion. EUS‐FNB was performed for preoperative histological diagnosis using a 22‐G FNB needle (Acquire, Boston Scientific Japan, Tokyo, Japan) (Figure 1b). An attempt was made to puncture the lesion from the duodenal bulb, but the mass could not be visualized, puncture was performed from the stomach to access the region. EUS‐guided sampling was performed in two separate sessions on different days. In the first session, one puncture with 30 strokes was performed. In the second session, three punctures were performed, with 30 strokes in each puncture, resulting in a total of four punctures and 120 strokes across both sessions. The procedure was terminated when an adequate amount of visible whitish core tissue was obtained. The pathology confirmed adenocarcinoma.

The preoperative clinical diagnosis was: Ph, TS1 (9.7 mm), cT1b, cCH0, cDU0, cS0, cRP0, cPV0, cA0, cPL0, cOO0, cN0, cM0, cT1b, cN0, cM0, and cStage IA (UICC eighth). The patient received two courses of neoadjuvant gemcitabine plus S‐1 (GS therapy: Gemcitabine 1000 mg/m^2^ div day1,8, S‐1 80 mg/day day1–14, triweekly). A subtotal stomach‐preserving pancreatoduodenectomy was performed, yielding the following pathologic diagnosis: Ph, ypTS1 (7 mm), int, INFb, ly0, v0, ne0, mpd0, pCH0, pDU0, pS1, pRP0, pPV0, pA0, pPL0, pPCM0, pBCM0, pDPM0, R0, P0, H0, CY0, pN0, cM0, Grade 1b, and ypStage IA (UICC eighth). The patient declined adjuvant chemotherapy and was followed postoperatively. Follow‐up CT scans were performed every 6 months for the first 2 years after surgery.

**FIGURE 2:**
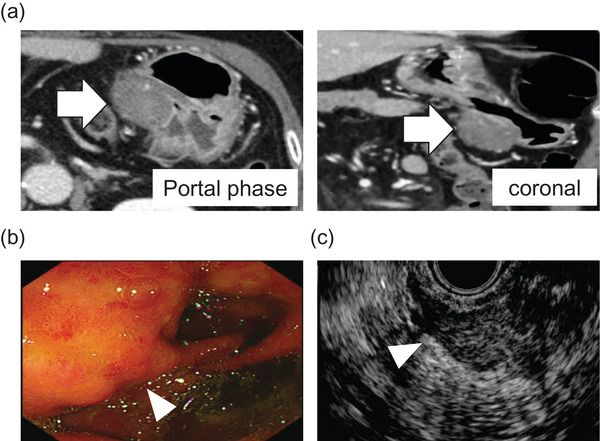
(a) CT scan at 2 years post‐preoperative for pancreatic cancer. Arrows (⇒) indicate that the mass appeared at the same site as the EUS‐FNB puncture, one to the primary pancreatic cancer, NTS was suspected. (b, c) Arrowheads (▷) indicate the mass in (b) esophagogastroduodenoscopy and (c) EUS. (b) Endoscopic images of the mass. (c) EUS‐FNB for the mass. CT, computed tomography; EUS‐FNB, Endoscopic ultrasound‐guided fine‐needle biopsy; NTS, Needle tract seeding.

Two years later, CT revealed a 30‐mm mass on the lesser curvature of the stomach with mildly enhanced, partially ill‐defined margins (Figure 2a). Laboratory tests showed only mild liver enzyme elevation without increased tumor markers (Table S1). Because the lesion developed along the prior EUS‐FNB puncture route to the primary pancreatic head lesion, NTS was suspected, and a tissue diagnosis was attempted.

On endoscopy, the lesion appeared as a submucosal tumor in the gastric antrum near the anastomosis (Figure 2b). EUS (GF‐UCT260) identified a 28.5‐mm, homogeneous, well‐demarcated hypoechoic mass arising from the muscularis propria (MP) layer, with internal linear hyperechoic strands and poor vascularity. EUS‐FNB was initially attempted with an FNB needle; however, because the lesion was extremely firm and could not be penetrated adequately, an FNA needle was used instead. Three sessions were performed using a 19‐, 22‐, and 25‐G needle (EZ Shot3 Plus; Olympus Medical Systems, Tokyo, Japan) (Figure 2c). However, the samples were scarce due to the extreme hardness of the lesion, and only fibrous tissue with minimal atypia was obtained, preventing a definite diagnosis.

**FIGURE 3:**
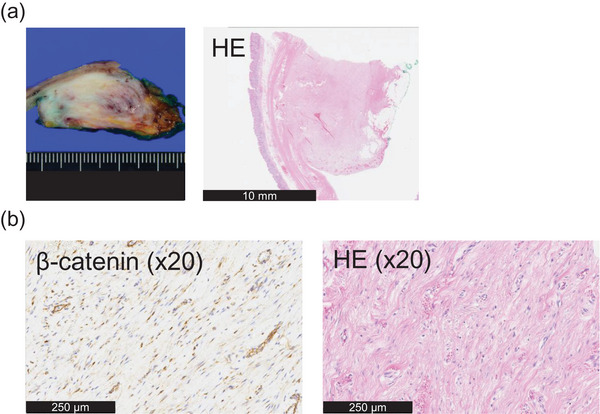
Pathological findings. (a) Macro image of the mass indicates white tone, indistinct borders, elastic‐soft lesion. (b) Hematoxylin and eosin staining and immunohistochemical staining show dense collagen deposition and nuclear β‐catenin (+), αSMA (‐), c‐kit (‐), and S‐100 (‐), leading to the diagnosis of desmoid fibromatosis.

Because NTS could not be ruled out, partial gastrectomy was performed for both diagnostic and therapeutic purposes. The resected specimen measured 3.0 × 2.8 × 1.5 cm and appeared as a white, poorly demarcated, elastic‐firm mass. The mass originated from the MP of the stomach and was located protruding toward the serosal side (Figure 3a and Figure S1). Hematoxylin and eosin staining showed dense collagen deposition without a capsule and proliferation of spindle‐shaped cells with minimal atypia. Immunohistochemistry revealed nuclear β‐catenin positivity and negativity for α‐SMA, desmin, CD34, S‐100, and c‐kit, confirming a diagnosis of DF (Figure 3b and Figure S1).

Two years after surgery, neither pancreatic cancer nor DF recurrence has been observed. To determine whether the gastric submucosal tumor contained any cancerous components suggestive of NTS, and to assess whether a DF component was present at the primary pancreatic cancer site, targeted amplicon sequencing was performed on both lesions.

**FIGURE 4:**
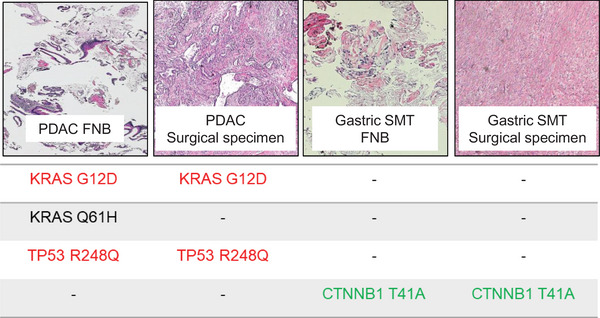
The results of gene mutation analysis using targeted amplicon sequencing for each specimen.

Targeted sequencing detected *KRAS* and *TP53* driver mutations in the primary pancreatic cancer tissue, whereas no such mutations were found in the gastric tumor. Conversely, a *catenin beta 1 (CTNNB1)* mutation (p.T41A; c.121A>G), characteristic of DF, was identified in the gastric lesion but not in the primary pancreatic cancer tissue (Figure 4). These findings indicated that the gastric submucosal tumor represented a DF rather than NTS, likely induced by mechanical stimulation related to EUS‐FNB.

## 3 Discussion

DF is a rare soft‐tissue neoplasm that, despite being histopathologically benign, demonstrates locally invasive growth and a tendency for recurrence, placing it clinically at the interface between benign and malignant tumors [3]. DFs are broadly classified into FAP‐associated types, which are driven by *APC* mutations, and sporadic types, which are often triggered by mechanical stimulations such as surgery or trauma and typically harbor *CTNNB1* mutations [4]. Histologically, DFs are characterized by uniform bundles of elongated spindle cells and dense collagen deposition with nuclear β‐catenin positivity on immunostaining. After transgastric EUS‐FNA/FNB of pancreatic body/tail lesions, NTS most commonly develops in the gastric wall along the puncture route; in a nationwide survey, 97.4% of pancreatic cancer‐associated NTS lesions were located in the gastric wall, with a median interval of 19.3 months from EUS‐guided tissue acquisition to detection [5]. On standard endoscopy, the most typical finding is a subepithelial/submucosal tumor‐like elevation, usually in the posterior gastric wall and often with a preserved mucosal surface. On EUS, these lesions are commonly described as hypoechoic intramural masses within the gastric wall. However, their diagnosis can be challenging due to the absence of specific imaging and pathological findings [6].

In the present case, NTS was initially suspected because the lesion arose precisely along the EUS‐FNB puncture route. Nonetheless, preoperative differentiation between DF and NTS was difficult, as the lesion lacked specific imaging and histological findings of DF.

An association between DF development and prior surgery or trauma has long been recognized, particularly in the abdominal wall and extremities, where it is referred to as cicatricial fibromatosis [7]. A small number of postoperative intra‐abdominal DF cases have been reported, including cases occurring after radical resection of pancreatic cancer [8, 9]. Previously described DFs after pancreatic cancer surgery arose near the root of the mesentery in proximity to the operative field. In contrast, the DF in our case developed at a gastric MP site exactly consistent with the EUS‐FNB puncture path. Although DF after EUS‐FNB has not been previously reported, a recent study on trauma‐associated sporadic DF identified punctate trauma (e.g., injections or trocar‐related injury) as a distinct category [10], supporting the concept that even limited focal injury can trigger clinically overt DF. It couldn't be completely excluded that the lesion developed incidentally as a postoperative complication of pancreatic surgery, but the fact that the lesion became evident only after EUS‐FNB and it remained confined to the gastric MP along the puncture route might make localized trauma from EUS‐FNB a biologically plausible immediate trigger.

In conclusion, we report a case of DF arising along the EUS‐FNB puncture tract that closely mimicked NTS. The relationship between radical pancreatic cancer surgery and DF formation, as well as the relationship between EUS‐FNB and DF, remains unclear. The present case highlights the importance of including DF in the differential diagnosis when a new mass appears along the EUS‐FNB puncture route following pancreatic cancer surgery.

## Author Contributions

**Hidemasa Kawabata**: conceptualization and supervision. **Kenji Takahashi**: advised and supervised. **Nobue Tamamura**: investigation and methodology. **Mishie Tanino**: investigation, methodology, and writing – review and editing. **Kazuya Koyama**: writing – original draft, writing – review and editing, and conceptualization. **Tetsuhiro Okada**: writing – review and editing.

## Funding

This study was supported by JSPS KAKENHI (Grant Numbers 21K07954) (to Kenji Takahashi).

## Conflicts of Interest

The authors declare a potential conflict of interest related to a collaborative research agreement with Olympus Corporation (to Mikihiro Fujiya) and Hitachi High‐Tech Corporation (Research funding) (to Yusuke Ono). The company had no role in the study design, data collection, analysis, interpretation, or manuscript preparation.

## Supporting information




**FIGURE S1** Additional radiologic and pathologic findings. (a) Macro image of the mass. (b) Immunohistochemical staining for αSMA and c‐kit.

**TABLE S1** Results of laboratory test. No evidence of elevated tumor markers.

## Data Availability

The data that support the findings of this study are available on request from the corresponding author. The data are not publicly available due to privacy or ethical restrictions.
