# Supplementary figures and images for: Desmoid Fibromatosis of the Gastric Wall Mimicking Needle Tract Seeding after Endoscopic Ultrasound‐Guided Fine‐Needle Biopsy for Pancreatic Cancer: A Case Report
Source: DEN Open. 2026 Apr 28;7:e70336. doi: 10.1002/deo2.70336 (PMC13122257; doi:10.1002/deo2.70336)

Figure. S1

(a)

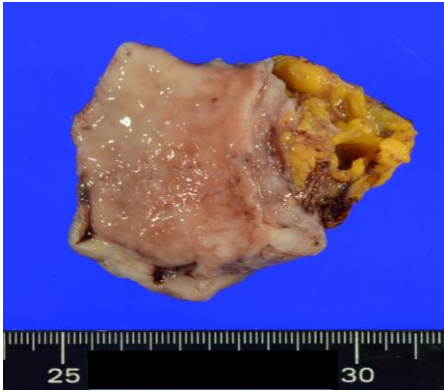

(b)

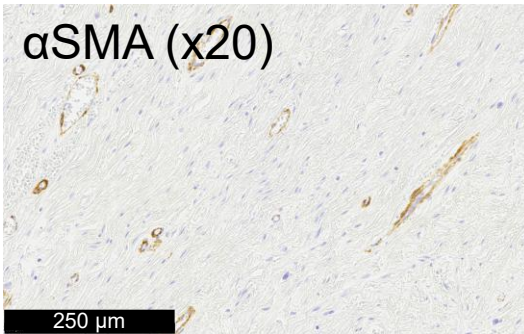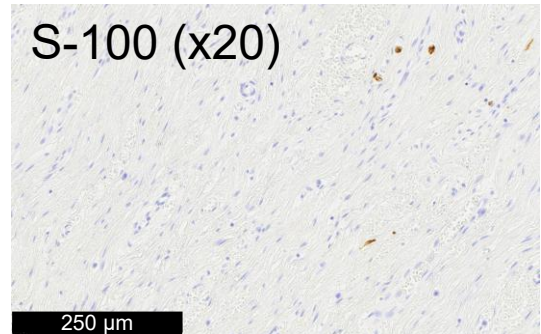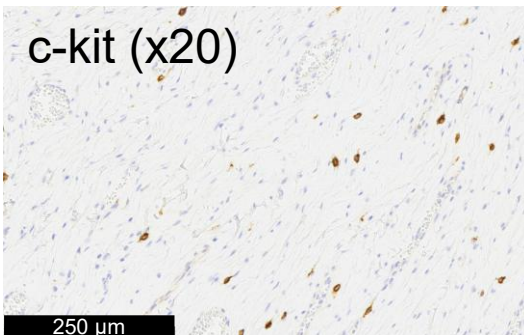

Supplement: Supplementary file 1 — FIGURE S1 Additional radiologic and pathologic findings. (a) Macro image of the mass. (b) Immunohistochemical staining for αSMA and c‐kit. [file DEO2-7-e70336-s002.pdf]
